# Single institutional outcomes of whole brain radiotherapy for metastatic melanoma brain metastases

**DOI:** 10.1186/s13014-021-01754-0

**Published:** 2021-02-08

**Authors:** Cecilia Jiang, Troy J. Kleber, Jeffrey M. Switchenko, Mohammad K. Khan

**Affiliations:** 1 Emory University School of Medicine, Atlanta, GA USA; 2 Department of Biostatistics and Bioinformatics, Rollins School of Public Health, Emory University, Atlanta, GA USA; 3 Winship Cancer Institute, 1365 Clifton Road NE, Atlanta, GA 30345 USA; 4 Department of Radiation Oncology, Emory University, Atlanta, GA USA

**Keywords:** Melanoma, Brain neoplasms, Neoplasm metastasis, Radiotherapy, Cranial irradiation

## Abstract

**Background:**

The management of melanoma with brain metastases (MBM) is increasingly complex, especially given recent improvements in targeted agents, immunotherapy, and radiotherapy. Whole brain radiation therapy (WBRT) is a longstanding radiotherapy technique for which reported patient outcomes and experiences are limited. We sought to report our institutional outcomes for MBM patients receiving WBRT and assess whether other clinical factors impact prognosis.

**Methods:**

A retrospective review of a single institution database was performed. Patients diagnosed with MBM from 2000 to 2018 treated with WBRT, with or without other systemic treatments, were included. Post-WBRT brain MRI scans were assessed at timed intervals for radiographic response. Clinical and treatment variables associated with overall survival (OS), distant failure-free survival (DFFS), local failure-free survival (LFFS), and progression-free survival (PFS) were assessed. Data on radiation-induced side effects, including radionecrosis, hemorrhage, and memory deficits, was also captured.

**Results:**

63 patients with MBM were ultimately included in our study. 69% of patients had 5 or more brain metastases at the time of WBRT, and 68% had extracranial disease. The median dose of WBRT was 30 Gy over 10 fractions. Median follow-up was 4.0 months. Patients receiving WBRT had a median OS of 7.0 months, median PFS of 2.2 months, median DFFS of 6.1 months, and median LFFS of 4.9 months. Performance status correlated with OS on both univariate and multivariable analysis. BRAF inhibitor was the only systemic therapy to significantly impact OS on univariate analysis (HR 0.24, 95% CI 0.07–0.79, *p* = 0.019), and this effect extended to multivariable analysis as well. Post-WBRT intralesional hemorrhage decreased DFFS on both univariate and multivariable analysis. Of patients with post-treatment brain scans available, there was a 16% rate of radionecrosis, 32% rate of hemorrhage, and 19% rate of memory deficits.

**Conclusions:**

Outcomes for MBM patients receiving WBRT indicate that WBRT remains an effective treatment strategy to control intracranial disease. Treatment-related toxicities such as intralesional hemorrhage, necrosis, or neurocognitive side effects are limited. With continued innovations in WBRT technique and systemic therapy development, MBM outcomes may continue to improve. Further trials should evaluate the role of WBRT in the modern context.

## Background

Melanoma is an aggressive malignancy with a propensity towards the brain, with brain metastases (BM) clinically diagnosed in 40–50% of metastatic cases and observed in up to 90% of autopsies [1]. Until recent treatment advances, patients with BM had a median survival of fewer than 5 months [2]. Although the prognosis for melanoma with brain metastases (MBM) has traditionally been poor, recent innovations in diagnostic imaging, systemic therapies, and radiation therapy have helped improve outcomes [3]. The role of whole brain radiotherapy (WBRT) in the modern context has become even more contentious.

Among the treatment options available to MBM patients, WBRT has played a prominent role ever since the 1990s, when prospective trials initially documented its benefits in treating all-comers with brain metastases with respect to local and distant intracranial control, overall survival (OS), and/or symptomatic improvement [4, 5]. This benefit has been noted especially for the management of single brain metastases in the post-operative setting [4, 5]. Since then, however, prospective data on WBRT has been limited, particularly in the MBM subset for whom tumors are likely to be more radioresistant. Also, WBRT is associated with unfavorable neurocognitive side effects that have been well-documented [6]. For example, a phase III trial on patients receiving WBRT showed a resultant decrease in the Hopkins Verbal Learning Test-Revised (HVLT-R) score from 7.04 points at baseline to 6.17 points at 24 weeks with WBRT [7]. When considering concurrent developments in stereotactic radiosurgery (SRS) and systemic therapies with improved intracranial penetrance, both the indications and outcomes for WBRT in the contemporary era cohort are even less clear, with some practitioners abandoning it altogether.

In 2019, results from the largest phase III trial evaluating WBRT specifically for MBM were reported. In this study, 207 MBM patients randomized to either WBRT or observation following local BM treatment showed no significant differences in 12-month intracranial control, OS, or global quality of life, suggesting no clinical benefit to WBRT [8]. However, only those with 1–3 BM were included, and there is still a paucity of data on the outcomes for patients with (a) higher intracranial burden and (b) a metastatic melanoma diagnosis [8]. Meanwhile, recent national database-driven studies suggest continued reliance on WBRT as a treatment option, with WBRT utilization rates quoted at > 85% in all-comers with BM [9, 10].

In our study, we evaluate our institutional experience with WBRT in the MBM population over the past 18 years, with a specific emphasis on patients with multiple BM. We report endpoints such as OS, local and intracranial control, and radiation-induced side effects, and we also compare outcomes for MBM patients who receive WBRT in the context of different multimodality treatment regimens to clarify the potential role of WBRT in this population.

## Methods

### Study design and treatment

This single-institution retrospective study was approved by the Institutional Review Board at Emory University. All adult (> 18 years old) patients who had received WBRT at Emory University from 2000 to 2018 were identified. Patients were excluded if they completed < 10 fractions of WBRT or did not have a diagnosis of metastatic melanoma. Patient characteristics such as age, gender, performance status, and systemic therapies were collected. Performance status was quantified according to the Eastern Cooperative Oncology Group (ECOG) and Karnofsky Performance Status (KPS) scales. The number of brain metastases at the initial time of BM diagnosis as well as immediately preceding WBRT were collected from brain MRI imaging reports. This variable was grouped into categories of 1–4 BM, 5–10 BM, or > 10 BM. Central nervous system (CNS) progression prior to WBRT was defined as an increase in the number of reported BM from the initial time of diagnosis to the time of WBRT.

All patients underwent computed tomography (CT) simulations with head immobilizations prior to WBRT. WBRT was delivered externally via opposed lateral fields, and the inferior border was set at the C1-2 vertebrae. Treatment was delivered with 6 MV photons. The receipt of additional therapies such as SRS, immunotherapies, or targeted therapies before or after WBRT was determined from patient charts. Concurrent ipilimumab/nivolumab was defined as dual ipilimumab and nivolumab administered on the same day. At our institution, all other oncologic therapies were paused for the duration of WBRT.

All follow-up imaging studies performed within 24 months after WBRT were reviewed to identify any local, intracranial, or extracranial tumor progression. The presence of post-treatment radionecrosis and intralesional hemorrhage was determined based on a review of radiology reports. Both radionecrosis and intralesional hemorrhage were defined solely based on radiologic criteria. Existing clinic notes during this period were also assessed for progression in memory deficits. If worsened memory issues were noted by the patient or physician in clinical documentation, this was considered progression in memory deficits. For patients without known dates of death, documentation from the last follow-up was used to ascertain the patient’s functional status and whether transition to hospice was discussed.

### Study endpoints

Endpoints of OS, distant failure-free survival (DFFS), local failure-free survival (LFFS), and progression-free survival (PFS) were calculated from the WBRT start date. OS was defined as the time to death from any cause. If the date of death was unknown, OS was defined as the time to the date during which transition to hospice was discussed or agreed upon. DFFS was defined as the time to the development of a new radiographically confirmed brain metastasis post-WBRT. Similarly, LFFS was defined as the time to the first radiographically confirmed growth in a pre-existing brain lesion post-WBRT, as documented by radiologists on imaging reports. Finally, PFS was defined as the time to distant failure, local failure, extracranial disease progression, death from any cause, or the discussion or agreement of hospice.

### Statistical analysis

Statistical analysis was performed using SAS 9.4 (Cary, NC) [11]. Descriptive statistics such as frequencies and percentages were generated for categorical variables such as performance status or the presence of extracranial disease, and means with standard deviations were generated for numeric variables such as age or WBRT dose. Survival distributions were estimated using the Kaplan–Meier method and compared using log-rank tests. Median, 6-month, and 12-month OS, PFS, DFFS, and LFFS were reported with 95% confidence intervals (CI). Univariate Cox proportional hazards models were fitted for OS, PFS, DFFS, and LFFS using covariates of interest as identified by the investigators. Model assumptions were checked and verified, and a *p*-value of < 0.05 was considered statistically significant. Multivariable Cox proportional hazard analysis was performed for OS and DFFS on select covariates of interest.

## Results

**Fig. 1:**
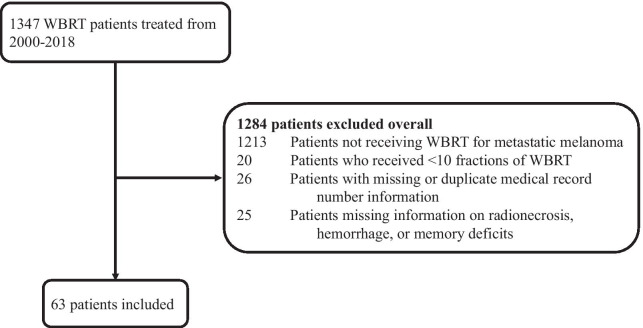
Diagram of patient selection and exclusion criteria

A total of 1347 WBRT patients were identified from treatment records at Emory University from 2000 to 2018. Of this group, 63 MBM patients were ultimately included in our study after excluding those who did not get WBRT for metastatic melanoma or were unable to complete a full course of WBRT. Figure 1 illustrates the selection and exclusion criteria. Of the 63 patients included, 39 patients (62%) were male, 24 patients (38%) were female, and the mean age was 54 years old (range 23–84, standard deviation 12.2). 27 patients (43%) had a KPS > 80. The decision to initially proceed with SRS or WBRT was made at multidisciplinary conferences that took into account performance status, systemic disease burden, and number and spatial distribution of BMs among other variables. Of the 23 patients (36%) who were immediately triaged to WBRT as opposed to salvage WBRT following SRS, the majority were found with > 5 BMs at the time of BM diagnosis. For all patients in our study, extracranial metastases were present in 43 patients (68%) at the time of WBRT. Eighteen patients (31%) had 1–4 BM, 32 patients (54%) had 5–10 BM, and 9 patients (15%) had > 10 BM at the time of WBRT. Also, the mean LDH level was 225 U/I (range 58–662, standard deviation 141) compared to the normal range of 12–246 U/I. 45 patients (71%) received a WBRT dose of 30 Gy (Gy) over 10 fractions, while the majority of the remaining 18 patients (29%) received 37.5 Gy over 15 fractions (13%) or 35 Gy over 14 fractions (8%). The other WBRT treatment plans were individualized but each included total RT dosage of at least 30 Gy (range 30–44 Gy). The majority of patients received WBRT along with one or more systemic therapy options, and no patients received a second course of WBRT. Salvage SRS only occurred in a minority of patients, as WBRT was more commonly used as salvage following SRS. A summary of patient characteristics before and after WBRT can be found in Table 1.

**Table 1:** Patient characteristics before and after whole brain radiotherapy

Variable	Number of patients	% Total
*Sex*		
Male	39	61.9
Female	24	38.1
*Year of diagnosis of BM*		
< 2011	52	82.5
≥ 2011	11	17.5
*Number of BM before WBRT*		
1–4	18	30.5
5–10	32	54.2
> 10	9	15.3
Unspecified	4	–
*CNS progression prior to WBRT*		
Yes	33	58.9
No	23	41.1
Unknown	7	–
*Extracranial metastases*		
No	20	31.7
Yes	43	68.3
*KPS*		
≤ 80	36	57.1
> 80	27	42.9
*ECOG performance status*		
0	14	22.2
1	33	52.4
2–3	16	25.4
*Temozolomide*		
No	30	47.6
Yes	33	52.4
*Interleukin-2*		
No	53	84.1
Yes	10	15.9
*Interferon*		
No	44	69.8
Yes	19	30.2
*BRAF inhibitor*		
No	55	87.3
Yes	8	12.7
*MEK inhibitor*		
No	60	96.8
Yes	2	3.2
Missing	1	–
*Concurrent ipilimumab/nivolumab*		
No	61	96.8
Yes	2	3.2
*Post-WBRT radionecrosis*		
No	53	84.1
Yes	10	15.9
*Post-WBRT intralesional hemorrhage*		
No	43	68.3
Yes	20	31.7
*Post-WBRT memory deficits*		
No	51	81.0
Yes	12	19.0
*Dose of WBRT (Gy)*		
Mean	32.16	–
Median	30	–
SD	3.59	–
*Number of WBRT fractions*		
Mean	11.71	–
Median	10	–
SD	2.81	–
*LDH (before WBRT)*		
Mean	225	–
Median	179	–
SD	141	–
*Age at WBRT (years)*		
Mean	54.21	–
Median	55.45	–
SD	12.18	–

BM, Brain metastases; WBRT, Whole Brain Radiation Therapy; CNS, Central Nervous System; KPS, Karnofsky Performance Status; ECOG, Eastern Cooperative Oncology Group; Gy, Gray; LDH, lactate dehydrogenase

### Overall outcomes

**Fig. 2:**
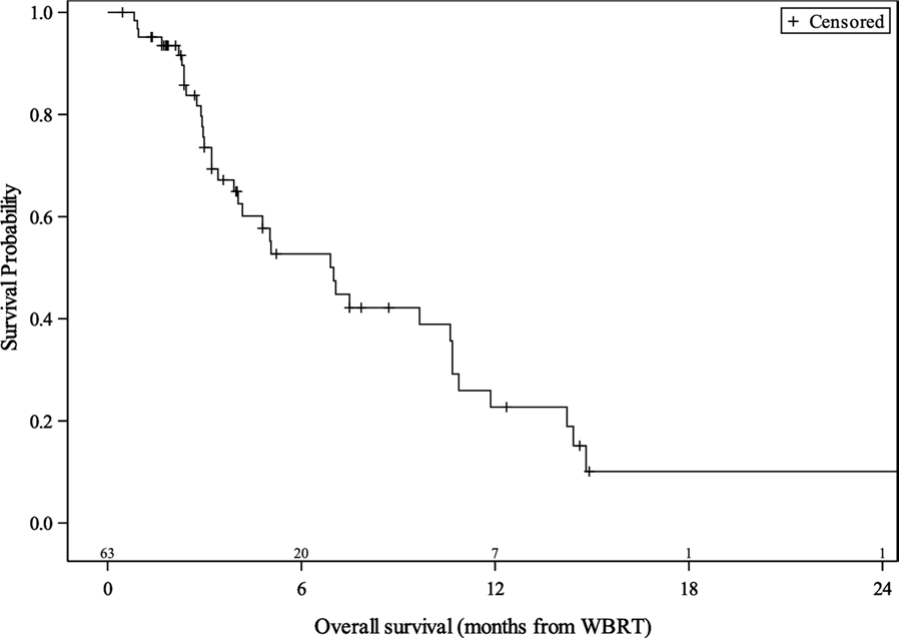
Kaplan–Meier overall survival curve for MBM patients treated with WBRT (n = 63). Kaplan–Meier curve for MBM patients treated with WBRT (n = 63) shows median survival of 7 months (95% CI 4–10.7), 6-month survival of 52.7% (95% CI 37.4–65.9), and 12-month survival of 22.7% (95% CI 10.6–37.5)

After WBRT, patients were followed for a median duration of 4.0 months. The median OS of the study population was 7 months with a 6-month and 12-month survival rate of 53% and 23%, respectively (Fig. 2). A univariate analysis was performed on covariates affecting survival. As expected, ECOG 1 (hazard ratio 3.66, 95% CI 1.44–9.30, *p* = 0.006) and ECOG 2–3 (HR 3.45, 95% CI 1.28–9.31, *p* = 0.015) patients had worse survival outcomes compared to ECOG 0 patients. This effect extended to multivariable analysis as well. Meanwhile, administration of BRAF inhibitors was associated with a statistically significant survival benefit on univariate analysis (HR 0.24, 95% CI 0.07–0.79, *p* = 0.019). This effect also extended to multivariable analysis. Table 2 illustrates the univariate and multivariable analysis of factors associated with OS.

**Table 2:** Univariate and multivariable analysis for factors influencing overall survival

Covariate	N	Univariate analysis		Multivariable analysis	
		Hazard ratio (95% CI)	*p*-value	Hazard ratio (95% CI)	*p*-value
*Sex*					
Male	39	0.79 (0.40–1.58)	0.504		
Female	24	–	–		
*Year of diagnosis of BM*					
≥ 2011	11	0.47 (0.19–1.17)	0.105		
< 2011	52	–	–		
*Number of BM before WBRT*					
> 10	9	0.94 (0.26–3.42)	0.930		
5–10	32	0.50 (0.23–1.09)	0.081		
1–4	18	–	–		
*CNS progression prior to WBRT*					
No	23	0.89 (0.39–2.04)	0.784		
Yes	33	–	–		
*Extracranial metastases*					
Yes	43	0.81 (0.42–1.58)	0.543		
No	20	–	–		
*KPS*					
> 80	27	0.52 (0.25–1.08)	0.080		
≤ 80	36	–	–		
*ECOG performance status*					
2–3	16	3.45 (1.28–9.31)	**0.015**	2.98 (1.06–8.40)	**0.039**
1	33	3.66 (1.44–9.30)	**0.006**	3.71 (1.34–10.27)	**0.012**
0	14	–	–	–	–
*Temozolomide*					
Yes	33	1.77 (0.89–3.48)	0.101		
No	30	–	–		
*Interleukin-2*					
Yes	10	0.49 (0.17–1.41)	0.187		
No	53	–	–		
*Interferon*					
Yes	19	0.79 (0.37–1.70)	0.550		
No	44	–	–		
*BRAF inhibitor*					
Yes	8	0.24 (0.07–0.79)	**0.019**	0.25 (0.07–0.91)	**0.036**
No	55	–	–	–	–
*MEK inhibitor*					
Yes	2	0.25 (0.03–1.87)	0.178		
No	60	–	–		
*Concurrent ipilimumab/nivolumab*					
Yes	2	0.38 (0.05–2.85)	0.347		
No	61	–	–		
*Post-WBRT radionecrosis*					
Yes	10	1.06 (0.49–2.29)	0.881		
No	53	–	–		
*Post-WBRT intralesional hemorrhage*					
Yes	20	1.29 (0.59–2.83)	0.520		
No	43	–	–		
*Post-WBRT memory deficits*					
Yes	12	0.80 (0.35–1.85)	0.608		
No	51	–	–		

Statistically significant* p* values are bolded

BM, Brain metastases; WBRT, Whole Brain Radiation Therapy; CNS, Central Nervous System; KPS, Karnofsky Performance Status; ECOG, Eastern Cooperative Oncology Group

As for WBRT patients who received other systemic therapies such as MEK inhibitors, concurrent ipilimumab/nivolumab, interferon, interleukin-2, or temozolomide, none of these additional therapies led to significant differences in OS on univariate analysis (all *p* > 0.05). Notably, factors such as the year of BM diagnosis, number of BM immediately prior to WBRT, presence of extracranial metastases, or the presence of CNS progression prior to WBRT also did not show an association with survival (all *p* > 0.05). Additionally, patients who experienced WBRT-associated side effects such as radionecrosis, intralesional hemorrhage, or memory deficits did not experience worsened survival compared to those who did not (all *p* > 0.05).

**Fig. 3:**
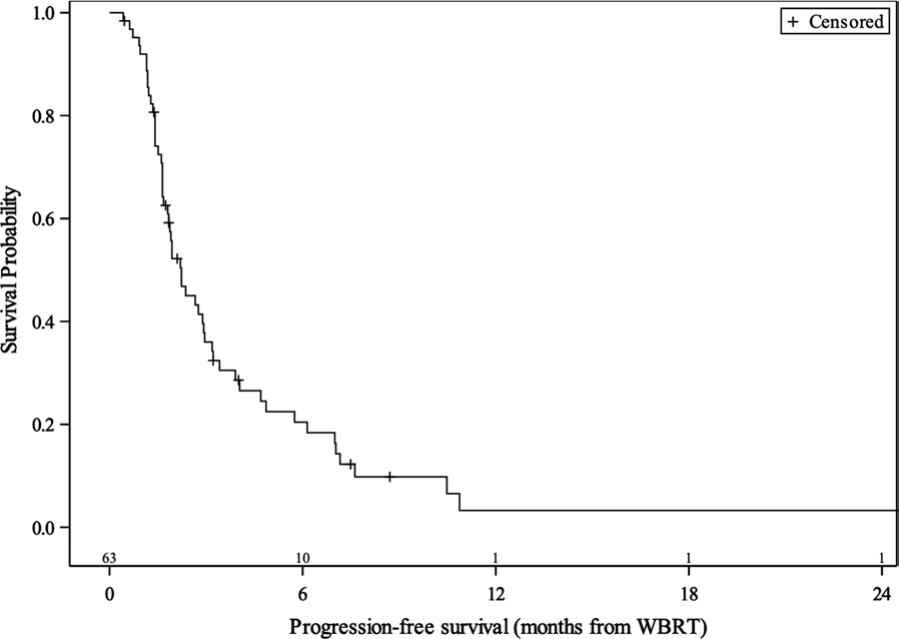
Kaplan–Meier progression-free survival (PFS) curve for MBM patients treated with WBRT (n = 63). Kaplan–Meier curve for MBM patients treated with WBRT (n = 63) shows median PFS of 2.2 months (95% CI 1.7–3), 6-month PFS of 20.4% (95% CI 10.9–32), and 12-month PFS of 3.3% (95% CI 0.3–13.4)

Meanwhile, median PFS was 2.2 months with a 6-month rate of 20% and a 12-month rate of 3% (Fig. 3). Patients with KPS > 80 at the time of WBRT had improved PFS (HR 0.53, 95% CI 0.30–0.94, *p* = 0.029), as did patients receiving multimodality therapy with BRAF inhibitors (HR 0.40, 95% CI 0.17–0.95, *p* = 0.038) on univariate analysis. Also, patients with ECOG 1–3 had worse PFS than patients with ECOG 0 (ECOG 1: HR 2.85, 95% CI 1.31–6.20, *p* = 0.008; ECOG 2–3: HR 1.86, 95% CI 0.80–4.33, *p* = 0.148), although the comparison between ECOG 2–3 and ECOG 0 was not statistically significant. Other variables such as treatment with interleukin-2, interferon, MEK inhibitors, BRAF inhibitors, or systemic therapies did not show an association with PFS, and neither did the presence of extracranial metastases, the number of BM, and the development of CNS progression (all *p* > 0.05).

### Intracranial outcomes

During the follow-up period, 10 patients (16%) developed radionecrosis, 20 patients (32%) developed intralesional hemorrhage, and 12 patients (19%) developed clinically significant memory deficits. Median LFFS was 4.9 months with a 6-month and 12-month rate of 43% and 19%, respectively. No systemic therapy, including concurrent ipilimumab/nivolumab, significantly affected LFFS on univariate analysis (all *p* > 0.05). The presence of CNS progression prior to WBRT also did not impact LFFS (*p* > 0.05).

Meanwhile, median DFFS was 6.1 months in patients receiving WBRT. DFFS was 52% at 6 months and 27% at 12 months. WBRT-related intralesional hemorrhage was associated with decreased DFFS on both univariate (HR 2.24, 95% CI 1.07–4.69, *p* = 0.033) and multivariable analysis (HR 2.76, 95% CI 1.25–6.07, *p* = 0.012). ECOG score of 1–3 also correlated with decreased DFFS when compared to ECOG 0 on univariate analysis (ECOG 1: HR 3.54, 95% CI 1.22–10.23, *p* = 0.020; ECOG 2–3: HR 2.22, 95% CI 0.72–6.84, *p* = 0.166), although the comparison between ECOG 2–3 and ECOG 0 was not statistically significant. Furthermore, this effect did not extend to multivariable analysis. No other variables, including extracranial metastases or the number of BM prior to WBRT, were identified as significant for DFFS. Table 3 illustrates the univariate and multivariable analysis of factors associated with DFFS.

**Table 3:** Univariate and multivariable analysis for factors influencing intracranial failure-free survival

Covariate	N	Univariate analysis		Multivariable analysis	
		Hazard ratio (95% CI)	*p*-value	Hazard ratio (95% CI)	*p*-value
*Sex*					
Male	39	0.90 (0.41–1.95)	0.780		
Female	24	–	–		
*Year of diagnosis of BM*					
≥ 2011	11	0.77 (0.31–1.90)	0.568		
< 2011	52	–	–		
*Number of BM before WBRT*					
> 10	9	0.71 (0.20–2.58)	0.603		
5–10	32	0.56 (0.25–1.27)	0.165		
1–4	18	–	–		
*CNS progression prior to WBRT*					
No	23	1.55 (0.67–3.59)	0.302		
Yes	33	–	–		
*Extracranial metastases*					
Yes	43	1.36 (0.62–2.98)	0.441		
No	20	–	–		
*KPS*					
> 80	27	0.50 (0.24–1.06)	0.072	0.53 (0.19–1.45)	0.214
≤ 80	36	–	–	–	–
*ECOG performance status*					
2–3	16	2.22 (0.72–6.84)	0.166	1.00 (0.21–4.69)	1.000
1	33	3.54 (1.22–10.23)	**0.020**	2.80 (0.79–9.95)	0.110
0	14	–	–	–	–
*Temozolomide*					
Yes	33	1.14 (0.54–2.39)	0.728		
No	30	–	–		
*Interleukin-2*					
Yes	10	0.94 (0.36–2.47)	0.905		
No	53	–	–		
*Interferon*					
Yes	19	1.10 (0.51–2.39)	0.805		
No	44	–	–		
*BRAF inhibitor*					
Yes	8	0.79 (0.30–2.10)	0.640		
No	55	–	–		
*MEK inhibitor*					
Yes	2	0.97 (0.23–4.16)	0.971		
No	60	–	–		
*Concurrent ipilimumab/nivolumab*					
Yes	2	2.14 (0.50–9.12)	0.305		
No	61	–	–		
*Post-WBRT radionecrosis*					
Yes	10	0.61 (0.21–1.76)	0.358		
No	53	–	–		
*Post-WBRT intralesional hemorrhage*					
Yes	20	2.24 (1.07–4.69)	**0.033**	2.76 (1.25–6.07)	**0.012**
No	43	–	–	–	–
*Post-WBRT memory deficits*					
Yes	12	0.65 (0.26–1.61)	0.354		
No	51	–	–		

Statistically significant* p* values are bolded

BM, Brain metastases; WBRT, Whole Brain Radiation Therapy; CNS, Central Nervous System; KPS, Karnofsky Performance Status; ECOG, Eastern Cooperative Oncology Group

## Discussion

In our series, we demonstrate favorable outcomes for MBM patients who undergo WBRT with a median OS of 7 months. This is much higher than expected for these patients, many of whom were offered WBRT as a last resort, after they had failed other options. Even with WBRT, survival for MBM patients has been exceedingly limited in the literature, with a recent retrospective series on 198 MBM patients documenting a median OS of 3.6 months with WBRT [12]. As a result, there has been a recent paradigm shift towards treating MBM with systemic therapies alone, such as concurrent ipilimumab/nivolumab, which have shown promising intracranial penetrance and survival outcomes in MBM patients. A recent phase II trial testing ipilimumab/nivolumab in 94 MBM patients showed a 12-month OS of 82% and an intracranial response rate of 56% [13]. However, it is important to note that only ECOG 0–1 patients with asymptomatic BM were included in this trial and that 76% of the patients had < 3 BM. Also, treatment toxicities were not insignificant, with a 55% rate of grade 3–4 adverse events, and one patient had a grade 5 adverse event. Given the pre-treatment characteristics of our patient population, which included 25% of patients with ECOG 2–3, 68% of patients with extracranial metastases, and 69% of patients with ≥ 5 brain metastases at the time of WBRT, our results indicate that WBRT still has potential to be a viable treatment option with comparatively minimal side effects, even for patients with unfavorable baseline characteristics.

There is limited contemporary data on WBRT outcomes in the MBM patient population. One retrospective series analyzed 61 MBM patients receiving WBRT with or without systemic therapies. In this study, where 92% of patients had ≥ 3 BM, patients were divided into cohorts depending on whether they received WBRT for newly diagnosed BM or intracranial disease progression. Both groups had a median OS of 3 months and the overall study population had a 59% rate of radiographically evident intracranial disease progression based on available post-WBRT MRI scans [14]. However, our series demonstrates more favorable outcomes with WBRT, with a median OS of 7 months, which did not differ significantly based on whether WBRT had been used for de novo or progressive BM. Six-month DFFS and LFFS were also 52% and 43%, respectively, indicating that the majority of our patients were experiencing more prolonged durations of CNS control with WBRT. These discrepant findings can be partially explained by the co-utilization of systemic therapies; in the Fuente et al. study, only 13% of patients received additional systemic therapy such as Ipilimumab or Temozolomide, compared to our patients, the majority of whom received systemic therapies. There is already emerging data that suggest that BRAF inhibitors are a potent radiation sensitizer, and low dose radiotherapy can enhance T cell infiltration within the tumor micro-environment [15]. Thus, future trials should evaluate the role of BRAF inhibitors and emerging immunotherapy agents in combination with WBRT.

Another retrospective study by Rauschenberg et al. also reported on WBRT outcomes in 92 MBM patients undergoing radiation and/or systemic therapies. In this study, the WBRT population experienced a similar median OS of 7.1 months [16]. Patients undergoing WBRT had a mean number of 5 BM, and the majority also had extracranial metastases. The addition of anti-PD1, anti-CTLA4, and BRAF inhibitor ± MEK inhibitor failed to significantly impact OS, although OS was notably above historical estimates of 2–4 months [16]. This was largely consistent with our study; except for combination therapy with BRAF inhibitors, which were given in 13% of patients, PFS and OS failed to significantly improve with the addition of other systemic therapy agents to the WBRT regimen. This could partially be explained by the limited number of patients receiving certain individual systemic therapy options, such as concurrent ipilimumab/nivolumab (3%).

The association between combination WBRT with BRAF inhibitors and improved outcomes in our study adds support to previous studies that identified BRAF mutant status as a positive prognostic factor [17]. A single-arm phase II trial of 172 MBM patients receiving Dabrafenib monotherapy resulted in an overall intracranial response rate of 35% in Val600Glu mutant patients with previously untreated or treated BM, suggesting good intracranial effect of this therapy [18]. Combining BRAF inhibitors with radiotherapy seems to further enhance this effect. In a retrospective pilot analysis of 12 MBM patients treated with Vemurafenib, the 3 patients who also received WBRT had either partial response (66%) or complete response (33%) intracranially. Their six-month OS of 92% was higher than what seen in our study, although SRS patients with limited BM were included in this analysis. The median number of BM in WBRT patients was 11 (range 6–12), suggesting that this response could be explained by the potential of BRAF inhibitors to serve as a radiation sensitizer [19]. This hypothesis has not fully been explored in the literature and warrants further investigation, especially in the era of more contemporary systemic therapy treatment options. Timing of WBRT and BRAF inhibitors was not explicitly stated in this study, but at our institution, it is our practice to pause BRAF inhibitors up to 3 days before WBRT to avoid skin toxicities such as cutis verticis gyrata, per consensus guidelines [20].

Our study also identified performance status as prognostic for OS and PFS. This is consistent with existing prognostic models such as the recursive partitioning analyses classes, disease-specific Graded Prognostic Assessment (ds-GPA), and melanoma marker GPA, which predict outcomes for patients with brain metastases [21–23]. However, other previously identified prognostic variables such as the number of brain metastases, age, or the presence of extracranial disease were not identified as prognostic in our analysis. This could be attributed to the limited size of our study population, changes in newer systemic agents, or more frequently timed brain MRI surveillance intervals allowing for earlier detection given the high proportion of patients with previously existing extracranial metastases. Notably, there were also no significant differences in OS, PFS, nor IFFS in our patients diagnosed with BM after 2011 compared to those diagnosed before on univariate analysis; although there are presumably more systemic therapies available to more recent patients, it is highly possible that newer patients who undergo WBRT have more aggressive disease that has been refractory to a wider arnamenterum of therapies.

Regarding WBRT-associated side effects, our patient series shows a 32% rate of post-treatment intralesional hemorrhage and a 16% rate of radionecrosis. With the development of multimodality treatment, there has been increasing concern over the possibility of a synergistically-motivated increase in radiation toxicities. However, the rate of radionecrosis in our patients, of whom > 50% also received systemic therapy, is consistent with the 4–24% rate of radionecrosis reported after radiotherapy alone [24, 25]. Nevertheless, the limited follow-up duration of our study precludes a complete assessment of radionecrosis incidence, which continues to occur over 12 months after radiotherapy [26]. Our institutional rate of post-WBRT intralesional hemorrhage is also consistent with a retrospective study performed by Klein et al., which reported a 31% rate of post-radiosurgery intralesional hemorrhage [27]. Reassuringly, neither the development of radionecrosis nor hemorrhage contributed to worsened OS or PFS, although the presence of intralesional hemorrhage significantly decreased DFFS. This correlation could be related in part to limitations in radiographic sensitivity in discerning between blood products and disease progression.

Notably, post-WBRT memory deficits were reported in under 20% of our patients. Due to particular concern over WBRT and associated neurocognitive toxicities, recent studies are evaluating the feasibility of an SRS-based approach for increasing numbers of MBM; for example, a recent study on 143 patients with 10 + BM reported a 96.8% local control rate in those treated with upfront SRS. However, new BM developed in 81.2% of patients, suggesting that DFFS remains a major limitation to SRS [28]. Our study results indicate that WBRT, even in combination with systemic therapy, can produce an advantageous median DFFS of over 6 months without inducing widespread neurocognitive deficits. Recent advances to WBRT, such as hippocampal-sparing contouring techniques or concurrent administration of Memantine, have shown promise in further sparing neurocognitive toxicities [29, 30]. An ongoing phase III trial comparing survival outcomes and quality of life in patients with 5–15 BM randomized to SRS or hippocampal-avoidant WBRT with Memantine should provide further information on these interventions (NCT 03550391).

Finally, our outcomes suggest that systemic disease progression continues to play a major role in outcomes. Previous studies have shown that death from extracranial progression is a predominant cause of death in MBM patients receiving WBRT [5]. In our analysis, neither the number of BM nor the presence of intracranial progression prior to WBRT impacted OS or PFS. Also, median DFFS and LFFS were notably above the median PFS of 2.2 months seen in our study. Taken together, this suggests that WBRT is helping control intracranial disease whereas systemic disease control continues to be a problem. Thus, the value of WBRT should not be discounted, despite the emergence of newer systemic options for MBM.

Limitations of this study include the small study size and retrospective single-institution nature of the study, which increases the likelihood of biases in factors such as patient selection. Another significant limitation arose from the lack of available death date records on all patients, which led to the designation of the date of transition to hospice or hospice discussions with providers as the date of death for those with otherwise incomplete information. As a result, the median OS and PFS experienced by our study population may be longer than reported, which would increase the favorability of WBRT. Also, the presence of memory deficits was identified through a review of patient charts, and information was lacking as to whether such deficits were attributed to WBRT or intracranial progression since formal neurological testing was not performed. Nevertheless, our study presents compelling evidence that MBM patients with multiple adverse characteristics can still benefit from WBRT. WBRT also seems to play a beneficial role in the multimodality setting, and future studies can further elucidate optimal treatment strategies for these late-stage patients.

## Conclusions

We report a retrospective review of 63 MBM patients undergoing WBRT. Even though greater than 50% of our patients at the time of WBRT had KPS < 80, and more than 60% had 5 or more brain metastases, we see 23% surviving at 12 months with reasonable toxicity. This warrants additional investigation in future trials, especially as it relates to concomitant use of emerging immunotherapy and targeted agents.

## Data Availability

The datasets used and analyzed during the current study are available from the corresponding author on reasonable request.

## Abbreviations

BM: Brain metastases
CI: Confidence interval
CNS: Central nervous system
CT: Computed tomography
DFFS: Distant failure-free survival
GPA: Graded prognostic assessment
Gy: Gray
HVLT-R: Hopkins verbal learning test-revised
KPS: Karnofsky performance status
LFFS: Local failure-free survival
MBM: Melanoma brain metastases
MRI: Magnetic resonance imaging
OS: Overall survival
PFS: Progression-free survival
SRS: Stereotactic radiosurgery
WBRT: Whole brain radiation therapy
